# Understanding internationalization variance within a sport brand: case study of the Sparta Prague ice hockey and football clubs

**DOI:** 10.3389/fsoc.2024.1459632

**Published:** 2024-10-25

**Authors:** William Crossan, Jan Šíma, Michaela Kaprálková, Tomáš Ruda

**Affiliations:** Department of Sports Management, Faculty of Physical Education and Sport, Charles University, Prague, Czechia

**Keywords:** Czech republic, eventification, figurational sociology, foreign ownership, globalization, sport migration

## Abstract

This article examines the internationalization of HC Sparta Prague (ice hockey) and AC Sparta Prague (football) by focusing on management decision-making. Using interviews with key personnel, it explores the varying levels of internationalization in these clubs, emphasizing their reliance on foreign players, the utility of foreign ownership, fan responses to foreign coaching, and marketing efforts toward foreigners. Football, being more global, is subject to ‘Europeanization,’ whereas ice hockey, centered in North America, is influenced by ‘Americanization’ and ‘eventification.’ Despite significant fan overlap and efforts at eventification, neither team has had success attracting Prague’s tourists and neither currently views it as an important market, instead focusing on the domestic Czech market. Both clubs have undergone various phases of ownership, from foreign investors to a return to Czech owners, reflecting broader social and economic changes in the region following the fall of communism. The article further discusses the influence of historical and cultural contexts on the acceptance and resistance to globalization influences, emphasizing the importance of preserving national identity and selectively integrating global elements within the modernization of sports. The study proposes that, despite the Sparta brand identity being heavily influenced by internationalization, it remains largely a Czech brand, and cultural sporting identity currently plays a more crucial role in resisting globalization than sport brand identity in Eastern Europe.

## 1 Introduction

In the summer of 2017, the AC Sparta Football team hired Italian coach Andrea Stramaccioni, who immediately brought 15 new foreign players into the team. The team’s owner, Daniel Křetínský, a global energy magnate who also owns a partial stake in the Premier League’s West Ham United, declared the hiring of a foreign coach to the Sparta team as the beginning of the “Internationalization” of the Sparta football team (AC Sparta Praha, 2017). It could be posited that the Sparta football team has long been considered an international team due to the presence of a Scottish coach as far back as 1912 (The Story of Johny Dick, 2014), however, this viewpoint was not shared by the team’s fans. Following the announcement of Stramaccioni’s hiring the news site idnes.cz conducted a survey of fans to gauge their opinions on the appointment. The results were overwhelmingly unfavorable, with 5,263 voters expressing opposition and only 1,908 expressing support (iDNES.cz, 2017). Stramaccioni’s tenure was short-lived, as he was dismissed after just 10 months due to disappointing results in both the domestic Czech football league and the UEFA Europa League. The internationalization project at Sparta was deemed a failure (Škuba, 2018), but at the same time, the process of globalization in the league appeared to be irreversible (Man, 2019).

## 2 Literature review

### 2.1 Cultural position

Football and ice hockey are far and away the primary sports in the Czech nation, with the largest numbers of active players, fan bases and media coverage. Czechoslovakia was playing these two games globally virtually from their inception, joining the governing body of football (FIFA) in 1904, and of ice hockey (IIHF) in 1908. AC Sparta Prague won the inaugural Mitropa Cup, a precursor to the UEFA Champions League, in 1927, and Czechoslovakia was one of the founding members of UEFA in 1954. Meanwhile, ice hockey has served to bolster national pride with 44 IIHF World Championship medals (13 gold), and 10 Olympic medals (1 gold) between Czechoslovakia and the Czech Republic. Ice hockey also served as a source of source of cultural resistance against the Soviet regime during the years of communist rule (Soares, 2007). Not only did the Czechoslovak national team win six IIHF World Championships during the communist regime from 1948 to 1989, several times defeating the USSR team, but the HC Sparta team also experienced international success winning the famed Spengler Cup twice (1962 and 1963) and obtained top three finishes in the Champions Hockey league or its predecessor, the European Hockey League, on six occasions between 1939 and 2017.

Despite Czechoslovakia’s history of being an early adaptor to global sports flows, including welcoming foreigners who aided early success in both football and ice hockey (Crossan, 2024), modern Czechs and Czech sport have been more resistant to recent efforts of internationalization than many of their Western European counterparts. This resistance to globalization can also be framed as a slower commodification of globalization when examining Eastern Europe (Molnar, 2011; Szerovay et al., 2017). Western Europe underwent significant economic and political integration earlier than Eastern Europe, especially after World War II, with the formation of entities such as the European Union (EU). This integration has facilitated globalization or the flow of capital, labor, and cultural exchange, including sports. In contrast, Eastern Europe was under communist rule until the late 1980s and the early 1990s, which isolated it from the capitalist West and delayed its economic development and integration into the global market. The Czech Republic joined the EU in 2004, opening borders to trade and the free movement of labor. Several recent works help us understand that globalization indeed occurred in Eastern Europe during the Cold War, but the push and pull were not just going from the traditionally understood West–East and did not simply follow the obvious core, periphery, and semi-periphery flows of World Systems Theory (Mark et al., 2020; Mark and Betts, 2021). Figurational theory (Dunning et al., 2004; Elias, 1978), developed by sociologist Norbert Elias, can help explain why Eastern European sports have been more resistant to globalization than Western European sports. This theory emphasizes the importance of understanding social processes, historical development, and interdependencies among groups to analyze societal changes. In the context of sports globalization, we can examine factors such as foreign sport ownership, sport migration, and the pursuit of global fanbases.

### 2.2 Foreign sport ownership

The English Premier League (EPL) is a prime example of how Western European sports have embraced globalization. Many EPL clubs are owned by foreign investors, such as Manchester City (owned by the Abu Dhabi United Group) and Chelsea (formerly owned by Russian oligarch Roman Abramovich). These investments have brought substantial capital, enhancing the league’s global appeal and competitive standards. However, fans have not always been in favor of foreign ownership (Coombs and Osborne, 2012; Williams and Hopkins, 2011), investment is commonly made for profit motives rather than the good of the club (Marin and Lee, 2020; Millward, 2011), and club history and tradition are frequently ignored (Berning and Maderer, 2020).

Foreign ownership is less prevalent in Eastern European clubs. Regulatory environments, economic instability, and political factors deter foreign investors (Rohde and Breuer, 2017). For instance, well-known Eastern European clubs such as CSKA Moscow or Dinamo Zagreb primarily remain under local or national ownership, limiting their financial capabilities and global reach. This is typical of many clubs in Eastern Europe, including the majority of Czech football and ice hockey teams. However, there are notable exceptions, such as Slavia Prague football, which was owned by the Chinese investment group Sinobo Group from 2015 to 2023. This foreign investment helped elevate the club’s financial status and competitive edge within the domestic league and European competitions. Prior to this, Sparta ice hockey was owned by the Anschutz Entertainment Group, an American company that bought six European ice hockey clubs, in addition to already owning the NHL Los Angeles Kings. This ownership brought substantial capital and international exposure before they sold it back to Czech owners in 2012, however these foreign owners remain exceptions rather than the rule in Czech sports.

### 2.3 Player migration

Football leagues like the EPL, Spain’s La Liga, and Italy’s Serie A search for and attract top talent from every corner of the world due to their financial power and global visibility. These high-profile players bring not only quality, but also global marketability to these leagues (Darby, 2013). Similarly, the NHL attracts top ice hockey players from the countries of Finland, Russia, Sweden, and the Czech Republic, with stars like Russian Alexander Ovechkin and Czech David Pastrňák again boosting the league’s competitive standard and international appeal (Crossan, 2020). The EPL is currently composed of 64% non-English players from 68 nations, while the NHL has 28% foreign players from 18 nations, illustrating that ice hockey has a much smaller global reach.

Eastern European leagues have struggled to attract and retain top talent. Instead, they often serve as feeder leagues, with talented Eastern European players moving to the West for better opportunities and higher wages (Rohde and Breuer, 2017). For example, Croatian star Luka Modrić was developed by Dinamo Zagreb before being sold to Tottenham Hotspur and eventually to Real Madrid, illustrating what many have termed talent drain (Agergaard, 2017; Marques, 2024).

### 2.4 Global fandom

European football clubs actively pursue global fanbases through various strategies including international tours, social media engagement, and broadcasting deals (Ekinci et al., 2022; McCarthy et al., 2022; Sullivan et al., 2022). For instance, Manchester United is estimated to have 1.1 billion fans worldwide, while FC Barcelona has over 500 million. Manchester United has even gone so far as to establish a museum in China to attract and engage fans (Yang, 2017). Many clubs regularly play preseason friendly matches on foreign soil, such as in the United States and Asia, to build their global brands. Additionally, they market foreign players back to their home nations to enhance their international appeal (Chiba, 2012; Madichie, 2009). Fans from around the world travel to attend games and take stadium tours, generating significant revenues from merchandise sales and media rights. Similarly, the NHL seeks to build a global fanbase by hosting games in Europe, such as the NHL Global Series, where teams play regular-season games in cities like Stockholm and Prague. They also broadcast their international stars, such as Ovechkin, back to Russia and Pastrňák back to the Czech Republic, significantly boosting their marketability in these countries. In 2018 the NHL began to strategically schedule certain games in the mid-afternoon in North America, ensuring that European viewers could watch them during primetime, thereby increasing the league’s international viewership and engagement (Berglund, 2020).

In contrast, Czech football and ice hockey clubs have primarily localized fanbases with limited global outreach (Crossan and Ruda, 2019). Economic constraints, less competitive leagues, and late internationalization efforts hinder their ability to market themselves globally. While Sparta football and Sparta ice hockey have strong local followings, their global presence is minimal compared to the top Western European football clubs or the NHL. This is despite significant numbers of international players, and Prague being a popular tourist destination. These clubs play only a few matches abroad when they can advance into European leagues and do not engage in extensive international marketing campaigns. Efforts to attract foreign fans or revenue through international broadcasting deals are limited, making it challenging to build substantial global fanbases.

### 2.5 Sparta brand and history

The Sparta Prague Football Club (AC Sparta), founded in 1893, quickly became a powerhouse in both domestic and European football, amassing numerous league titles and continental honors. Simultaneously, the Sparta Prague Ice Hockey Club (HC Sparta), established in 1903, built its own legacy of triumph, securing multiple national championships and international trophies. During the communist era from 1948 to 1989, the Sparta brand operated as a unified entity encompassing football and ice hockey, as well as various other sports, such as athletics and handball. Each sport was managed independently yet under the same Sparta umbrella. With the collapse of communism in Czechoslovakia in 1989, the clubs transitioned to privatized, independently managed entities while retaining their brand identity.

Sparta hockey became a joint-stock company in 1995 and was initially acquired by Czech auto racer Antonín Charouz, who later sold a controlling stake to the American investment group Anschutz Properties in 1999. As previously mentioned, Anschutz also owned stakes in the NHL’s Los Angeles Kings as well as the MLS’s LA Galaxy. Since 2019, Sparta hockey has been owned by the Kaprain Investment Group, with Czech investor Karel Pražák holding 100% of the stock. Similarly, Sparta football was privatized in 1992 when businessman Petr Mach purchased it from the state. It changed hands several times before coming under the ownership of J&T Private Equity Group in 2004, with Czech billionaire Daniel Křetínský as the primary investor and club president. Křetínský is also a major shareholder and director of the Premier League’s West Ham United. J&T is a joint Czech and Slovak investment group.

Despite this historical unity and shared name, Sparta hockey and Sparta football now operate as separate corporate entities. Each club focuses on maximizing fan engagement, cultivating sponsorship relationships, and leveraging broadcasting revenues within a limited yet segmented Czech market. The ACS Sparta football and HCS Sparta ice hockey teams have been central to national pride for many in the Czechoslovak and Czech populations, producing a high percentage of national team players and serving as launching pads for athletes to reach elite international leagues. Additionally, they are consistently ranked at the top of their respective national leagues.

Both Sparta clubs have ventured into international markets through foreign ownership but have since returned to Czech hands. Both teams import foreign players in order to be competitive in both domestic and international competitions. Efforts to build a significant global fanbase are still in progress. Despite having robust local followings, their international presence remains modest compared to that of the top Western European clubs. The fanbase intersection is notable, with one-third of Sparta hockey season ticket holders also holding football season tickets, and one-fifth of football season ticket holders attending hockey games.

## 3 Research objective

Globalization brings both benefits and challenges to cultures and sports teams. While sports have the unique ability to unite people across cultural divides, they also accentuate and amplify cultural differences. The Czech nation, with its distinct and evolving culture, strives for both preservation and uniqueness, a dynamic also reflected in its primary sports: ice hockey and football. These two sports possess both global and local cultures, and significance. Consequently, cultural diversity has become an increasingly important topic in the world of sports, with globalization—or internationalization—sometimes being commodified and at other times resisted to preserve and advance specific sporting cultures.

Globalization in sports has frequently been framed through the lenses of Europeanization (Brand et al., 2013; Weber, 2021), Americanization (Carlsson et al., 2022; Jackson, 1994), and eventification (Batuev and Robinson, 2018; Richelieu and Webb, 2021). Europeanization manifests in the dominance of European countries in top-tier football leagues and the governance of global football by organizations such as FIFA, headquartered in Zurich. This phenomenon positions Europe as the central hub for football excellence and administrative control, influencing the management of sports worldwide. Americanization, exemplified by the global proliferation of McDonald’s and American sports commodities such as the NHL, signifies the influence of American cultural and business practices worldwide. The NHL exemplifies Americanization by underscoring the prioritization of spectacle over pure sport performance. The NHL, with its predominantly North American teams, illustrates how American sports leagues and their marketing strategies, including early games for European audiences, have shaped global sports consumption. This trend is evident in the NHL’s emphasis on entertainment value, such as the extravagant All-Star Games and the outdoor Winter Classic, which transform games into grand spectacles. Furthermore, expansion of the NHL into non-traditional ice hockey markets, like Las Vegas with the Golden Knights and Arizona with the Coyotes, illustrates the league’s strategy to embed the sport within broader entertainment cultures, prioritizing market proliferation and audience engagement over traditional ice hockey strongholds (Crossan, 2020). Lidström and Carlsson (2022) outline how the predominance of the NHL lead to the Americanization and eventification of ice hockey in Europe. Eventification describes the transformation of sports into spectacular events to take advantage of larger audiences than those traditionally drawn to a particular sport or team (Richelieu and Webb, 2021). In football, this is seen in the grandeur of tournaments such as the FIFA World Cup and the UEFA Champions League, while in ice hockey, it is evident in the NHL’s Winter Classic and the Champions Hockey League. Both sports leverage eventification to enhance their global appeal, drawing fans into highly marketed celebratory experiences. Yet researchers have also found limits to the eventification of football, attributed primarily to preservation of cultural traditions (Carlsson and Backman, 2023; Hognestad, 2013).

This article explores various perspectives on internationalization within Czech culture, focusing on two sports under one brand: Sparta ice hockey and Sparta football. By examining adaptation, resistance, and the sporting context, this study highlights the disparity in the role of globalization between these two Sparta teams. Using secondary data and qualitative interviews, we seek to investigate the similarities and differences within three domains of globalization: migration, ownership, and fandom for Sparta ice hockey and Sparta football.

## 4 Methods

This study employed a qualitative approach through semi-structured interviews with key personnel from two prominent sports clubs in Prague, Czech Republic: the AC Sparta Prague football club and the HC Sparta Prague ice hockey club. The aim was to explore the variance in internationalization strategies between these two clubs under the shared brand identity of Sparta Prague. The sample consisted of six respondents, three from each club, strategically selected to provide a comprehensive view of the clubs’ internationalization processes. The interviewees included top management, marketing directors, and sports managers responsible for strategic direction, communication strategies, and recruitment and integration of foreign players and coaches.

The interviews were structured to address four main areas of internationalization within the clubs: the recruitment, selection, and integration of foreign players; the presence and impact of foreign coaches and support staff; strategies for engaging both local and international fans; and the implications of foreign ownership on club strategies and operations. Each interview lasted approximately one hour and was conducted either in person or via video conferencing. Interviews were recorded with the consent of the participants to ensure accurate transcription and analysis.

The collected data were analyzed using thematic analysis, which involved several stages: transcription of the interviews, coding to identify significant statements and themes, grouping codes into broader themes, and interpreting these themes in the context of the research objectives. Codes were derived through an inductive approach, where patterns and recurring concepts were identified directly from the data rather than being imposed by pre-existing theories. To ensure the consistency and accuracy of the coding process, two independent researchers coded a subset of the data and compared their results. Inter-rater reliability was ensured through regular discussions and consensus meetings between coders to resolve any discrepancies and refine the coding framework. Measures to ensure the reliability and validity of the study included triangulation, member checking, and peer review. Triangulation involved comparing data from different respondents to identify common patterns and discrepancies, member checking entailed sharing preliminary findings with respondents for feedback, and peer review involved independent researchers reviewing the analysis process.

By employing this methodology, this study aims to provide a nuanced understanding of how internationalization is manifested and managed within the AC and HC Sparta Prague clubs. It highlights the unique challenges and strategies associated with each sport under the shared brand identity of Sparta Prague, offering insights into the complexities of balancing local loyalty with globalization pressures and opportunities.

## 5 Results

*“Sparta exists in an international environment, not only through our participation in European cups but also through the fact that the player market is free, and Sparta competes with other clubs around the world. We compete for players, for talent to be competitive, for sponsors, for fans, and our competition is global.”—ACS*

*“We approach internationalization only in terms of strengthening our squad so that we can meet the highest goals. We expect to reach the finals every year. We expect to play in the Champions Hockey League every year. We need the Czech nation to watch us play all three series [EHL regular season, EHL playoffs and Champions Hockey League] and we need foreign players to do that.”—HCS*

### 5.1 Player migration strategies

**Figure 1:**
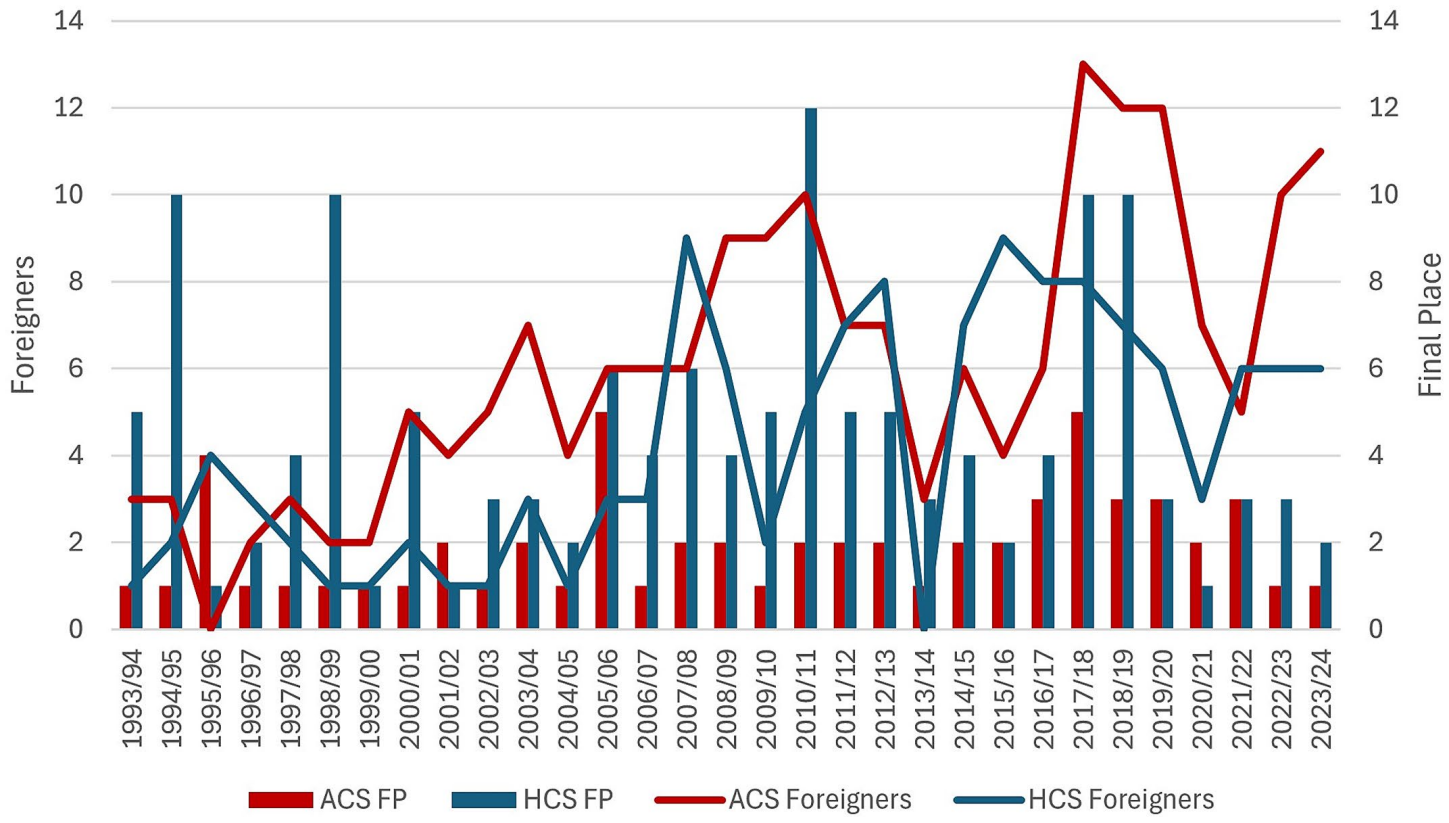
Final place and foreigners used. FP = Final Place which is indicated on the right axis.

From Figure 1, it is evident that while the Czech Republic is stronger on a global level in the sport of ice hockey, the Sparta football team has consistently outperformed the Sparta ice hockey team, with only three seasons in which ice hockey finished better. Both teams have steadily increased in their usage of foreigners over the 31 years represented here, but again Sparta football has consistently used more foreigners than Sparta ice hockey. This is also significant in light of the differences in roster sizes. The EHL allows teams to have 22 skaters on their roster for a game, but the average roster size for the 31 years examined was 35 players per team, with 13.5% of all roster spots being taken by foreigners. In contrast, the Fortuna football league allows teams to have 25 players on their roster for the season, of which 18 can be selected per game. For the 31 years examined, 14.5% of football roster spots were occupied by foreigners. Both Sparta football and ice hockey have consistently been at the top of their leagues in performance but also in usage of foreign players.

Both teams repeatedly stated that they bring in foreign players in order to be competitive in both the domestic league and international competitions. Specifically, they choose foreign players, whether in ice hockey or football, because they have a specific role, or post, on the team to fill, and there are currently no Czech players available to fill those roles. Both teams noted that the very best Czech players are playing outside the country, either in the NHL in the case of ice hockey, or in more prestigious leagues such as those in Western Europe in the case of football. Thus, these best Czech players are not currently available to HC or AC Sparta. Both teams stated they are more likely to look to foreign players when they know coming into the season that they will be playing in the UEFA Champions League (football) or the Champions Hockey League. This level of international play demands of them not only higher quality players, but more experienced players. And this level of player experience is a critical dividing point between ice hockey and football. Additionally, HC Sparta emphasized that when the team heads into the playoffs and needs to strengthen their roster foreign players take on increased importance. This is only possible in ice hockey as their transfer window is open longer and the nature of the sport is such that the playoffs essentially re-start every team from zero in terms of points. In football, playoff points are added to the points already earned during the season. That said, team managers emphasized the need at this stage of the season to not disrupt team chemistry, which is riskier in the employment of foreign players.

The average age of HC Sparta’s foreign imports over the last five seasons was 30, while AC Sparta had an average import age of 24. This data alone tells us much about both the differing philosophies of foreign player usage and the global positions of these two sports in the Czech nation.

*“We, as a club, are not a final destination. We are the club which brings in a player, raises his value, develops him, and sends him on. We actually want players who want to move away from us, who want in those 2-3 years to do something for us, they improve, their value goes up and they go to a better club for better money or better competition. This is the mentality that we must have. If we should become a destination for them, for the players, that they do not want to move on from us, that changes the situation completely, but we are not a destination. We never will be.”—ACS*

*“The best Czech players are already playing in the NHL, and we, along with the other extraleague teams are all fighting for the rest of them. We have an established system, based on the table value of players, where basically if a Czech player is not under contract, then he is the property of his club and it is very difficult to negotiate with other clubs. So most of the time it is easier to bring in a foreign player, and most of the players who come to the Czech Republic, or the one we want, have already come through some European leagues, if not the NHL, and have some experience.”—HCS*

These two quotes express the differing philosophies of Sparta football and ice hockey, with each philosophy rooted in the global position of the sport for the nation. Czech ice hockey consistently ranks among the top six in the world, thus contributing 30–80 players to the NHL each year over the last 25 years. Consequently, the EHL not only produces excellent young ice hockey players but also serves as a very competitive league as players move past their prime. As ice hockey is contested at an elite level in far fewer countries than football, the competitive balance of top European ice hockey leagues is very high, with many teams able to win the title in any given year. As each team has potential to produce young players who could reach the lucrative NHL, the league has established a system to reward teams for developing these valuable assets. This means young, talented Czech ice hockey players change hands infrequently inside the nation, and to field a competitive team one must bring in experienced, already developed foreigners. This system is exacerbated by the NHL’s draft system and Canada’s extensive junior league system (CHL), to which Czech is consistently the biggest contributor and serves as a feeder league to the NHL (Crossan, 2024). Thus, HC Sparta is pushed to internationalize, primarily in terms of player migration, simply to remain competitive within the domestic EHL.

In contrast, AC Sparta, shown in Figure 1, consistently finishes in the top three positions in the domestic Fortuna League (FL). The top three teams in the league have not only been consistently occupied by AC Sparta, SK Slavia, and FC Viktoria Plzen, but very few Czech players have made it out to the Big 5^1^ without going through these three teams (Crossan and Riedl, 2023). Thus, AC Sparta seeks to win the domestic FL each year, but does so knowing that their players, both Czech and foreign, are looking to keep developing and moving up the European football pyramid.

Thus, the dynamics of global positioning and player migration not only affect the typology of foreigners coming into each Sparta team but also the destinations where they land after taking off the Sparta jersey. Tables 1, 2 present the foreign imports and exports of both Sparta franchises over the last five seasons.

**Table 1:** Sparta foreign player imports 2019/20 to 2023/24.

Player name	Nationality	Age acquired	From team	Season
ACS Sparta Football				
Andreas Vindheim	Norway	23	Malmö FF (SWE)	2019/20
Martin Minchev	Bulgaria	19	Cherno More (BUL)	2020/21
Dominik Holec	Slovakia	26	MŠK Žilina (SVK)	2020/21
Dávid Hancko	Slovakia	23	Fiorentina (ITA)	2021/22
Casper Højer	Denmark	26	Aarhus GF (DEN)	2021/22
Kaan Kairinen	Finland	24	Lillestrøm (NOR)	2022/23
Lukáš Haraslín	Slovakia	26	Sassuolo (ITA)	2022/23
Asger Sörensen	Denmark	26	1.FC Nuremberg (GER)	2022/23
Qazim Laci	Albania	26	AC Ajaccio (FRA)	2022/23
Markus Solbakken	Norway	23	Viking (NOR)	2023/24
Angelo Preciado	Ecuador	25	KRC Genk (BEL)	2023/24
James Gomez	Gambia	21	AC Horsens (DEN)	2023/24
Indrit Tuci	Albania	23	NK Lokomotiva (HRV)	2023/24
Victor Olatunji	Nigeria	23	FC Slovan Liberec	2023/24
HC Sparta Ice Hockey				
Milan Jurčina	Slovakia	36	Nürnberg Ice Tigers (GER)	2019/20
Ville Koistinen	Finland	37	ERC Ingolstadt (GER)	2019/20
Matúš Sukeľ	Slovakia	23	HC Slovan Bratislava (SVK)	2019/20
Maxim Žukov	Russia	22	HC Dukla Jihlava	2021/22
Maxim Matuškin	Sweden/Russia	30	Admiral Vladivostok (KHL)	2021/22
Július Hudáček	Slovakia	33	Dinamo Riga (KHL)	2021/22
Genadij Stoljarov	Russia	35	Meran (ITA)	2021/22
Oleg Pogorišnyj	Russia	27	Jugra Chanty-Mansijsk (RUS)	2021/22
Erik Thorell	Sweden	29	SV Zug (SUI)	2021/22
Dominik Graňák	Slovakia / Czechia	39	HC Plzeň	2022/23
Olavi Vauhkonen	Finland	33	SaiPa (FIN)	2022/23
Stefan Warg	Sweden	32	KooKoo (FIN)	2022/23
Gustaf Thorell	Sweden	33	HC Škoda Plzeň	2022/23
Ostap Safin	Czechia /Russia	23	Bakersfield Condors (AHL)	2022/23
Zack Kassian	Canada	33	Arizona Coyotes (NHL)	2023/24
Nicolas Beaudin	Canada	24	Laval Rockets (AHL)	2023/24
Aaron Irving	Canada	27	HC Davos (CHE)	2023/24
Jani Lajunen	Finland	33	Örebro HK (SWE)	2023/24
Josh Kestner	USA	29	Rukko Rauma (FIN)	2023/24

Teams without a country are Czech. For ice hockey, we chose to list the international league that the team plays in rather than the country.

**Table 2:** Sparta player exports 2019/20 to 2023/24.

Player name	Nationality	Age sold	To team	Season
AC Sparta Football				
Eldar Civic	Bosnia	23	Ferencváros (HUN)	2019/20
Bogdan Vătăjelu	Romania	26	CS U Craiova (ROM)	2019/20
Gol-Gol Mebrahtu	Australia	28	Puskás AFC (HUN)	2019/20
Zinedin Mustenaganic	Bosnia	21	FK Sarajevo (BOS)	2020/21
Tal Ben Haim	Israel	30	M. Tel Aviv (ISR)	2020/21
Martin Frýdek	Czechia	28	FC Luzern (CH)	2020/21
Costa Nhamoinesu	Zimbabwe	34	Kerala Blasters (IND)	2020/21
Georges Mandjeck	Cameroon	31	Waasland-Beveren (BEL)	2020/21
Semih Kaya	Turkey	29	Malatyaspor (TUR)	2020/21
Guélor Kanga	Gabon	29	Red Star Belgrade (SRB)	2020/21
Uros Radakovic	Serbia	27	Arsenal Tula (RUS)	2021/22
Benjamin Tetteh	Ghana	24	Malatyaspor (TUR)	2021/22
David Moberg Karlsson	Sweden	27	Urawa Reds (JAP)	2021/22
Libor Kozák	Czechia	32	Puskás AFC (HUN)	2021/22
Srdjan Plavšić	Serbia	25	Slavia Praha	2021/22
Lukáš Štětina	Slovakia	30	Spartak Trnava (SVK)	2022/23
Adam Hložek	Czechia	19	Bayer Leverkusen (GER)	2022/23
Dávid Hancko	Slovakia	24	Feyenord (NED)	2022/23
Michal Sáček	Czechia	26	Jagiellonia (POL)	2022/23
Matěj Pulkrab	Czechia	25	SV Saundhausen (GER)	2022/23
Matěj Hanousek	Czechia	30	Ankaragücü (TUR)	2023/24
Florin Niță	Romania	36	Gaziantep (TUR)	2023/24
Tomáš Čvančara	Czechia	22	Bor. M’Gladbach (GER)	2023/24
James Gomez	Gambia	22	Odense BK (DEN)	2023/24
Casper Højer	Denmark	28	Rizespor (TUR)	2023/24
Adam Gabriel	Czechia	22	Midtjylland (DEN)	2023/24
Ondřej Čelůstka	Czechia	34	BodrumFK (TUR)	2023/24
Milan Heča	Slovakia	32	Slovácko	2023/24
Matin Minchev	Bulgaria	22	Rizespor (TUR)	2023/24
HC Sparta Ice Hockey				
Marek Ďaloga	Slovakia	30	Dinamo Riga (KHL)	2019/20
Erik de la Rose	Sweden	26	VIK Västeras HK (SWE)	2019/20
Steven Delisle	Canada	29	Löwen Frankfurt (GER, 2. liga)	2019/20
Zach Sill	Canada	31	Kölner Haie (GER)	2019/20
Adam Polášek	Czechia	28	Tappara Tampere (FIN)	2019/20
Kevin Klíma	USA/Czechia	23	Mountfield HK	2020/21
Ville Koistinen	Finland	38	HPK Hämeenlinna (FIN)	2020/21
Jeremie Blain	Canada	28	Mountfield HK	2020/21
Roberts Bukarts	Latvia	30	HC Severstal Čerepovec (KHL)	2020/21
Filip Chlapík	Czechia	24	Ottawa Senators (NHL)	2020/21
Matúš Sukeľ	Slovakia	25	HC Verva Litvínov	2021/22
Alexander Salák	Czechia	35	Djurgårdens IF (SWE)	2021/22
Lukáš Rousek	Czechia	23	Buffalo Sabres (NHL) / Rochester Americans (AHL)	2021/22
Matěj Machovský	Czechia	29	Dimano Riga (KHL)	2021/22
Andrej Kudrna	Slovakia	31	HC Verva Litvínov	2021/22
Genadij Stoljarov	Russia	36	Jugra Chanty-Mansijsk (VHL)	2022/23
Július Hudáček	Slovakia	34	Barys Astana (KHL)	2022/23
Nino Tomov	Bulgaria	18	Drummondville Voltigeurs (QMJHL)	2022/23
Maxim Matuškin	Sweden/Russia	31	Tappara Tampere (FIN)	2022/23
Oleg Pogorišnyj	Russia	28	Tambov (VHL)	2022/23
Olavi Vauhkonen	Finland	34	Jokerit (FIN)	2022/23
Gustaf Thorell	Sweden	34	BIK Karlskoga (SWE)	2022/23
Ostap Safin	Czechia/Russia	24	Lada Togliatti (RUS)	2023/24
Erik Thorell	Sweden	32	Frölunda HC (SWE)	2023/24
Stefan Warg	Sweden	34	Vienna Capitals (AUT)	2023/24
Martin Jandus	Czechia	25	Kärpät Oulu (FIN)	2023/24
David Tomášek	Czechia	27	Färjestad BK (SWE)	2023/24

Teams without a country designation in the To team column are Czech teams.

As shown in Table 1, the transfer to AC Sparta was an improvement for all 14 players in terms of the level of play or playing time at the time of transfer. Some of the sending clubs are at AC Sparta’s level (Sassuolo, KRC Genk), while some have a higher level (Fiorentina), but the players who transferred from those clubs to ACS typically did not have stable positions in the team. When we compare Player Value at the time of transfer and current player value, out of 14 foreigners who transferred to ACS, only three have lower values now. Therefore, we can conclude that three out of 14 did not improve as a result of their time representing AC Sparta.

Of the 19 hockey players who transferred into HC Sparta (imports), six players improved their situation, seven transferred from comparable clubs, and six took a step down. For those whose situation worsened, this was due to the end of their engagement in the NHL, AHL, or KHL. In all cases, the players had lost their status in their previous clubs, and in five cases, they were middle-aged or older players, with only Ostap Safin returning from the AHL at age 23. The six players who improved their situation by coming to HCS came from the German, Italian, or Czech leagues. Of particular interest are the two Slovak players Sukeľ and Hudáček who came to HCS from the KHL. In both cases, they transferred after their teams left the KHL, either for financial reasons or as a protest of Russia’s invasion of Ukraine. Information on the costs incurred for ice hockey transfers to the club is not publicly accessible; therefore, we did not evaluate increases or decreases in ice hockey player values.

In terms of exports, we do not see quite the difference in average age that we saw in imports, with ACS exporting players on average at age 27 and HCS at 29. However, the differing global positions of these two sports can be specially highlighted here, with football exports going to 17 different nations, including seven to Turkey, while sending only Tomáš Čvančara and Adam Hložek to teams in the Big Five. Conversely, HCS exports are exclusively to the handful of ice hockey power nations or within the Czech league itself.

Players who transferred out of ACS are more difficult to generalize than those imported. The most common departures where players who did not catch on in Sparta. These players were either released for free or for less than they were acquired. However, the transfers of Hancko, Hložek, Čvančara and more recently Krejčí put the overall balance into clear positive numbers. Significantly, the majority of players who have been sold for larger sums are all Czech players rather than foreigners. This was confirmed in the AC Sparta interviews, where the respondent said that foreigners are purchased primarily to fill current competitive needs, while Czechs are purchased for their development potential. Despite AC Sparta’s ability to remain at the top of the domestic league, the majority of their foreign transfers from the last five seasons have failed economically or sportingly. The few exceptions, such as the transfer of Slovak player David Hancko to Feyenoord for 8.3 million, after acquiring him for 2.5 million, have proven sufficient to keep ACS overall financial balance positive. Interestingly, currently, Hancko is valued at 35 m, so one can question whether AC Sparta let him go too soon.

Table 2 lists 27 ice hockey players who left the HCS club, which is similar to the 29 football players who went out in the top half of the table. During the five seasons examined, 20 foreign skaters left the club, and only three transferred to a higher-quality league, specifically the KHL. The remaining 17 players mainly transferred within the Czech league to lower-level clubs (4 players), to lower quality leagues in Europe (5 players) or returned to their home leagues (8 players). Characteristically, Sparta tends to bring in older, experienced combative foreigners and thus these players at the end of their careers are unlikely to move up to higher level leagues. In total, seven Czech players left HC Sparta for foreign contracts between the 2019/20 and 2023/24 seasons. Two of these players moved to the NHL, one to the KHL, and Smejkal’s move to the Finnish league can also be considered as an improvement for the player due to a highly lucrative contract. This transfer was particularly lucrative for the player himself. The remaining three transferred to clubs of similar quality in prominent ice hockey nations.

### 5.2 Foreign coaches

Senior executives provide distinctly different responses to the question of hiring foreign coaches. For the executive from AC Sparta, it was *“crucial to bring someone from outside the Czech market to disrupt the established order.”* By contrast, the top manager of HCS opposed hiring a foreign coach, citing language barriers and the potential for communication misunderstandings with certain players.

Interestingly, the language barrier, or rather its elimination, is one of the reasons why ACS explicitly prefers hiring foreign coaches for its coaching staff. Not only is there a foreign coach currently leading the A team, replacing another foreign coach, but the B team, playing in the second league, also intentionally includes several foreigners in its coaching staff. *“This forces young players to communicate in English, and become accustomed to working in an international environment with players and coaches of* var*ious nationalities. It excellently prepares them for their future career steps.”—ACS.*

Nevertheless, the AC Sparta executive emphasized that ACS is a Czech club and wants to profile itself as such. *“From the beginning, we wanted a combination of Czechs and foreign coaches in the coaching staff, ideally ensuring that there is always a Czech in every position. This way, the Czech coach remains when the foreign staff leaves. There is some interaction, the Czech coach acquires the foreign coach’s know-how, and we retain this knowledge locally.”-ACS.*

The statement from ACS’s top manager aligns with the club’s long-term concept. The club’s management is convinced that having a foreign coach contributes to its diversity and cultural enrichment. Players and staff can learn different ways of thinking and working in a multicultural environment, which can be highly valuable in preparing players for their international careers. Thus, the presence of a foreign coach can increase player motivation and the overall professionalism of the club.

The contrast with HCS, which had foreign coaches in the past, was surprising and was expressed by all three interview respondents. *“The foreign coach has it harder in terms of perception. Players cannot express their feelings in English. Even if they understand the hockey instructions. Even though the language has shifted over the last 20 years, I do not think it’s a good idea to have a foreign coach in Czech conditions. It is just terribly difficult.”—HCS* These responses are surprising, given that young HCS players have aspirations to reach the NHL, many of whom achieve these dreams. Additionally, the majority of the foreigners HCS imports have already played for multiple international teams, including the NHL.

### 5.3 Fan responses

The specifics of each player were mentioned frequently when discussing fans’ perception of foreign players. The individuality and specificity of each situation apply to both reactions to foreign players and coaches. In addition, both Sparta football and hockey representatives emphasized that performance plays a crucial role in how foreign players or coaches are perceived.

*“I would say that nowadays we have the most foreign players we ever had. There are quite a lot of them, however, nobody perceives it negatively because the sporting results are great.”—ACS.*

The Sparta football representative also pointed out that performance matters, but with a visible shift in recent years, stating: *“When I came to Sparta and there was a player who wasn’t necessarily better than the rest of the squad, it might be harder for him to be perceived positively than for the Czech players. From that time, we became a “European” team and club, so this issue is not a thing anymore.”—ACS.*

This shift in perception echoed another ACS respondent, who was convinced that if there was a survey today that asked fans their favorite players, there would not be much difference between foreigners and players who grew up in the club’s academy.

HCS also believed that a shift in fan perception had occurred, but that shift was more related to the quality of foreigners being brought in and the results: *“Sparta was also perceived for many years to have bought many foreigners. It did not rely enough on its academy players, but rather on retired 2^nd^ and 3^rd^ line foreigners who basically come to Prague because it’s attractive as a destination. Now I think that’s changed and it’s been perceived positively that most of the foreigners have either NHL experience or have some experience coming from good leagues, and we are winning, which always helps.”—HCS.*

Both teams emphasized that building an internationalized environment goes hand in hand with treating foreigners as equal to domestic players within the club. This starts with the management and the coach, and both marketing managers understood their own roles in shaping fan perceptions. The HCS representative mentioned that the Americanization of marketing is related to the use of players for marketing and PR purposes on a larger scale; however, there were no differences between use of Czech and foreign players. The ACS respondent mentioned storytelling as one of the main marketing tactics—that means showing the staff and players in different situations and environments, raw footage of training, etc. This approach then helps fans focus on the actual characteristics and personalities of foreigners within the club, where their nationality is just one part of it.

The HCS respondent explained that fans expect every foreigner to be a star, but often the foreign player has not been brought in to be a first line player. *“I can go through the roster and tell you what role we bought every foreigner for.” – HCS* Thus, it is the team’s job not only to explain to the market the past experience that the foreign player brings, but also to establish clear expectations of the player to the fans.

Multiple respondents expressed that cultural stereotypes and transfer prices or remuneration put pressure on foreign players. In the case of hockey, fans sometimes prefer players from so-called “hockey nations,” nonetheless, they also expect great performance from them, and the reactions might be more negative when the results aren’t so good, or it seems like the player just wanted to finish their career in Prague comfortably without much ambition to help the team.

*“In general, for the Czech fans, it is more interesting when we buy a Canadian, American, Swedish of Finnish player rather than a German.”—HCS.*

The Sparta football representative used an example of a Czech football player bought by rival Slavia Prague for a high price, stating, *“I would say that what makes expectations bigger is not the nationality but rather the price. Foreign players tend to be more expensive; however, this is not always the case. Look at Slavia Prague buying Zima for 100 m CZK [4 M €], he’s getting a hard time from the fans. A Czech player, who loves the club, and the transfer price made it more difficult for him. So, if there is something important then it is usually the price of remuneration more than the nationality.” ACS.*

In terms of internationalization, making the fans more understanding and breaking the barriers of nationality, a football representative also stated: *“We have a huge responsibility toward the whole society because the fans follow us. And when we make some decisions and set some standards, we can get a huge number of people to identify with it and practice the same values” ACS.*

### 5.4 Foreign ownership and sponsorship

While foreign ownership affected both ACS and HCS in the past, representatives from both teams expressed extreme contentment with their current Czech investors, primarily in terms of market aspirations and performance goals. This is significant, given the investment potential of many foreign investors. Multiple respondents stated that Czech investors will act more responsibly in terms of the brand and tradition of the club based on their connection to the Czech people. The respondents felt that foreign investors were only concerned about the economic aspects of the club. The current Czech investors are willing to invest sufficient capital in both HCS and ACS to allow the teams to pursue their stated goals, and it was said that neither team returns any investment to their owners.

In terms of AC Sparta, while ownership is in the hands of the Slovak J&T portfolio, the club president and responsible person within J&T is Křetínský who aside from his previously mentioned involvement with West Ham United, is influenced by his other global assets: *“Sparta today is already, you could say, global because our owners are actually operating in markets all over Europe and they co-own the Royal Mail in the UK and they own a lot of assets in Italy, in Holland, in Germany, in Belgium and so we have actually been under a lot of influence of globalization as a club today, so our owner or our owners are operating in different global markets and those influences are already there.”—ACS.*

As has already been stated, HC Sparta views internationalization primarily through the use of foreign players. Thus, their Czech owner is willing to pay for whatever player they need, whether Czech or foreign, which is the most important aspect.

It is significant to add here that most of AC Sparta’s income comes from the international market, generated primarily through its participation in European cups and player sales, while HCS relies primarily on the domestic market with monies invested by the owner and shares of domestic broadcasting revenue. As one of the respondents from HC Sparta stated in comparison, *“Sparta football, even though they play well and they are not a disgrace Europe… But you saw that when they played Liverpool, they are not exactly a European giant. [2024 Europa League round of 16 Sparta lost both games with Liverpool 6:1 and 5:1] We have an advantage in this. It’s true European hockey and football are incomparable in terms of financial rewards, but on the other hand, like [HC] Sparta in the Champions Hockey League or the Spengler Cup. I dare say we are one of the top clubs in Europe.”—HCS.*

A third type of foreign investment in the Sparta teams comes through sponsorship revenue, where significant differences emerge between football and ice hockey. The most important partners for Sparta football are international companies. The team’s general partner is the Greek company Betano, while its main partners include the German companies adidas and T-Mobile. It is only at the third level of sponsorship, known as official partners, that the majority of sponsors are Czech companies. In contrast, most of the sponsorship revenue for Sparta ice hockey comes from domestic sources. The general partner is the Czech bookmaker Fortuna, and all main partners are based in the Czech Republic. While there are a few international companies among Sparta ice hockey’s official partners, they remain in the minority.

### 5.5 Expanding the fan base

The managers of both clubs expressed interest in attracting international fans, displaying logic in line with the potential to increase revenue primarily through eventification. However, neither Sparta club currently considers this a priority. Neither club is willing to invest significant effort in targeting tourists who visit Prague in large numbers every year for its historical landmarks. Yet both clubs have taken steps in this direction and are planning to expand their fan bases internationally in the future. The potential in terms of available stadium capacity, facilities, merchandising, and social media is outlined in Table 3.

**Table 3:** Sparta foreign fan options.

	ACS – Sparta Football	HCS – Sparta Ice Hockey
World league ranking	13-17	5-6
Nations represented on team	Total: 18	Total: 9
	Albania, Australia, Bulgaria, Cameroon, Czech Republic, Ecuador, Finland, Gabon, Georgia, Ghana, Nigeria, Norway, Romania, Serbia, Slovakia, Sweden, Turkey, Zimbabwe	Bulgaria, Canada, Czech Republic, Finland, Latvia, Russia, Slovakia, Sweden, USA
Average season attendance and stadium capacity fulfillment	2019/20–8,634–44%	2019/20–9,042–53%
	2020/21–3,285–17%*	2020/21–0–0%*
	2021/22–7,349–38%*	2021/22–13,200–77%
	2022/23–14,347–76%	2022/23–13,226–77%
	2023/24–17,084–93%	2023/24–13,743–80%
Stadium tours, Fan shops or museums	Stadium tour includes a tour of the museum, press center, locker rooms, dugout and playing field. Ends in the fan shop.	Only fanshop in the training arena.
Social media	Instagram: General, women’s + esport	Instagram: 61,6 K followers, at least 2 post every day, story highlights, bigger interaction than on Facebook
	General Instagram: 234 K followers, post at least 2 times a dayWomen’s Instagram: 13,3 K followers, post almost everyday Facebook: 277 K followers, post same as Instagram, less interactions	
		Facebook: 99 K followers, at least 2 post every dayYouTube: 13,5 K subscribers, 1,3 K videos, 2 times a week, approximately 1 K viewsTikTok: 32 K followers, 880 K likes, 2 times a weekSpotify: podcast, 3 episodes in 2020
	X: Main account: 69,7 K followers	
	YouTube: 79 K subscribers, once a week, around 40 K views
	Twitch: 12,6 K followers, live streams
	TikTok: 211,7 K followers, 3,9 mil. Likes

Data represents seasons 2019/20 to 2023/24; * attendance affected by the coronavirus pandemic; Sources: TeamForm League Ranking (teamform.com), Champions Hockey League Ranking (championshockeyleague.com), Transfermarkt (transfermarkt.co.uk), Livesport (livesport.cz), Fortuna liga (fortunaliga.cz), Hokej.cz (hokej.cz).

*“Sparta is strong on the international field, it will attract more fans from other regions, because the second club phenomenon is still quite common. We have my local one that we support, or the one in the country I live in, but then I also follow a club that I support in the European Cups because I like it in some way. And we as Sparta have a huge advantage here because Prague is a tourist city, very attractive.”—ACS.*

AC Sparta football currently faces a primary barrier in that local fan interest is so high that tickets sell out within hours or days of going on sale through their mobile application. Consequently, very few tickets are available through traditional sales channels that tourists might use. As a result, the marketing director of ACS does not see much value in heavily promoting ticket sales to tourists. Nevertheless, he noted that certain products are specifically marketed to international tourists, including items from the fan shop and stadium tours. *“Previously, we had one tour per week; now, we conduct 80 tours per month, with three to four tours daily. Approximately one-third of these tours are conducted in English.”* The ACS respondents viewed the promotion of merchandise and stadium tours as inadequate and planned to expand their marketing efforts and product offerings to the Prague city center and airport in the future.

The ACS stadium (Epet Arena) has a capacity of 18,887 seats and is relatively outdated and inadequate in many respects. Due to the increasing interest of fans in purchasing season tickets, Sparta plans to construct a new stadium with a capacity of 35,000 spectators, financed independently. Club managers have confirmed that with the opening of the new stadium, the situation will change dramatically and attracting international tourists will become a priority. This will allow them to offer tourists *“a modern sports arena experience in Central Europe, alongside the Prague Castle and Charles Bridge.”*

*“For us, the priority is to have our Czech fans there, to build that long-term relationship. Unlike the tourists who come here maybe once or twice in their lifetime. We see ourselves as a Czech club at the moment, with fans from all over central Czech, not just Prague. Expanding to include a foreign audience is not just about ticket sales, but also about keeping the same energy. It’s a kind of forward-looking vision. It’s a question of how feasible, how much Sparta can become a global club.”—ACS.*

HCS currently operates a modern ice hockey arena, the second largest in Europe, which hosted the IIHF World Championship in 2024. This event drew a record 797,727 spectators, averaging 12,464 per game. Attendance at Sparta hockey games has also been rising, with average attendance exceeding 13,000 spectators over the last three seasons, as indicated in Table 3. However, according to the marketing director of HCS, only a minimal number of these attendees are foreigners, approximately 200 for important games and as few as 40 for less popular ones. The revenue from these ticket sales is relatively low, prompting HCS to focus its marketing efforts on local fans, ideally those considering purchasing season tickets.

The capacity of O2 Arena for the top Czech hockey league is set at 17,220 seats, providing room to increase attendance further. Unlike AC Sparta, HCS sees current potential to attract more international fans. Although the top manager of HCS believes that tourists do not primarily come to Prague for hockey (unlike football tourists visiting England), both she and the marketing manager see a path to increasing the proportion of international fans over the long term. Certain initiatives aimed at this goal were planned but were not implemented due to the COVID-19 pandemic.

Both hockey managers envision a collaborative marketing strategy with football club ACS to target tourists. *“We would be one brand, each offering slightly different products.”* Significant activities in this area from both clubs are expected to commence closer to the opening of the new football stadium.

In terms of social media, both teams present content in Czech and English, and both teams post about Czech and foreign players. Nonetheless, the AC Sparta marketing representative expressed the current reality of Czech sports’ position on a European level well, *“We do not currently track foreign fans. We obviously see, on social media, the composition of foreigners. But you cannot really say that they are fans. Because when you play, say, Galatasaray Istanbul, you get 5,000 Turks coming to the club at the same time. But they are not your fans. Even if they come to your stadium, they are not likely to follow your club. To them we are still a Czech club.”—ACS.*

## 6 Discussion and conclusion

The two primary sports in Czech culture, ice hockey and football, play critical roles in the formation of Czech identity. These sports possess both global and local cultures and significance, reflecting the dynamic of the Czech nation striving for both preservation and uniqueness. This dynamic positions internationalization, examined through figurational theory, as experienced by the Sparta football and ice hockey clubs, as an increasingly important topic for Czech sports. We can extend this comparison, including its consequent tensions to other Eastern European nations, or to other contexts where we encounter Americanization versus Europeanization of sport. In the context of sports globalization, factors such as foreign sport ownership, sport migration, and the pursuit of global fanbases are critical in understanding these dynamics. Figurational theory (Dunning et al., 2004; Elias, 1978), emphasizing historical development, social processes, and interdependencies among groups, such as domestic leagues and international leagues, can help explain why Eastern European sport has been more resistant to globalization compared to Western European sport. Historical interdependencies and power dynamics help explain why football has been Europeanized, while ice hockey has become Americanized.

Football, a far more global game than ice hockey, remains centered in Europe and is subject to ‘Europeanization’ despite huge increases in foreign team ownership and repeated efforts to buy the absolute best players out of Europe. In contrast, ice hockey’s locus in North America subjects the game to ‘Americanization’ and increasing ‘eventification’. Sparta hockey, for instance, shows clear signs of influence from the NHL, adopting this model with far more eventified games than Sparta football. The nature of ice hockey, including the amount of stoppage time and competition format, is conducive to both Americanization and eventification. In terms of eventification, HC Sparta engages fans during commercial breaks with an interactive jumbotron, featuring not only the traditional kiss cam but also activities like fan-celebrity comparisons, loudness checks, and more. This past season, fan entertainers like Cameron Hughes also performed, energizing the crowd with his dancing during breaks in play. As part of the Americanization of the sport, referee helmet cams—primarily aimed at TV viewers—allow fans to listen in on discussions between players and referees. The influence of North American trends is also evident in the adoption of anglicisms by Czech coaches, players, reporters, and fans. Terms such as “skill,” “agility,” and “one-timer” have become commonplace in Czech hockey vocabulary. Additionally, Czech ice hockey has embraced rule changes inspired by the NHL, with the ‘Coach’s Challenge’ video review being one of the most recent innovations.

Even with five of the top six leagues, and four of the top six ice hockey nations, Europe has not been able to forestall the Americanization of ice hockey. The Sparta brand displays significant fan intersection, with 1/3 of all HC Sparta season ticket holders also owning AC Sparta season tickets. These fans have commodified the Americanization of ice hockey which still symbolizes their national identity, while also holding tightly to tradition at Sparta football games which are currently so full that there is no space for foreign fans.

While Czech fans rabidly follow the best Czechs in the NHL or the Big5, neither Sparta franchise has experienced or embraced a globalization strategy of reaching out to foreign fans from the nations from where their many imports hail. The fans embrace internationalization which bolsters national identity globally, while frequently still resisting globalization locally. Crossan et al. (2024) emphasized that in emerging Eastern European countries, such as the Czech Republic, Hungary, and Ukraine, the racial diversity within football clubs often exceeded that of their fan base. As a result, clubs frequently struggled with incidents of racist behavior from their supporters. With the growing reliance on foreign players and coaches, these clubs naturally became cultural leaders, not only in the fight against racism but also in embracing internationalization more broadly. Dunning emphasized that the social fabric and historical context of a sport in a society influence its approach to globalization (2004). This means that while internationalization brings new opportunities and resources, there is a simultaneous effort to maintain and celebrate what is unique about a nation’s sporting culture. Thus, AC Sparta has experienced success drawing foreign visitors to its stadium and museum, selling them merchandise as they exit, even while understanding that they are not, and probably never will be, Manchester United. Consequently, AC Sparta has so far preserved their event game atmosphere to cater to Czech fans, while taking advantage of the European trend of football stadium tours.

It is evident that the two clubs differ in their internationalization strategies. Notably, Sparta football is more competitive on the domestic level, while Sparta ice hockey is more competitive globally, often bringing in more experienced players with NHL experience. Both Sparta teams emphasize only using foreign players to fill roles which they cannot fill with equal quality for price Czech players. Elliott and Maguire (2011) emphasized that while the global movement of athletes, and coaches can enhance the quality and competitiveness of local leagues, it can also lead to tensions as communities strive to retain their unique sporting identities. As we saw at the beginning of this article AC Sparta fans were very resistant to a foreign coach until a foreign coach took them to the top of the domestic league table. Sparta hockey fans might be expected to be more interested in their import players given the numbers with NHL experience, yet they show less interest in foreign players compared to football fans. In any case, fans are more receptive when foreign players are of higher quality, which is more visible in football than in ice hockey given the global position Czech holds in these two sports. Fans also respond more positively when the marketing departments share clear expectations about the expected roles of these foreign imports, even following globalized marketing trends.

Meanwhile, despite HC Sparta outperforming AC Sparta in international competition, it is ACS which profits significantly from its international player transfers and participation in these European leagues. In this regard the Europeanized sport has outperformed the Americanized sport in terms of global capital flows. Thus alongside recent research (Mark et al., 2020; Mark and Betts, 2021) which explains Eastern Europe’s slower commodification to globalization outside of late arrival of outside capital and World Systems Theory, we can posit that commodification cannot be explained without taking into account local–global scope and interdependencies. The European international cup competitions in football, such as the UEFA Champions League and Europa League, have much more media coverage and provide more finances to football than to ice hockey competing in the Champions Hockey League. Football has developed into a far more global game than ice hockey, thus multiplying revenue generating possibilities for football, which Eastern European teams are keen not to be left out of. But the size of the nation, and the reality that the best Czech players will leave home in both sports to play in the top global leagues, means that local teams in both sports need foreign players to compete in European leagues. If they only competed domestically, they would not need foreign players, but for football, such an internationalization backstep would mean a significant loss of revenue.

The clubs increasingly rely on foreign players while varying in their philosophies of migrant acquisition. The average age of imported players in ice hockey stands notably higher at 30.4 years, reflecting the sport’s reliance on seasoned professionals. In contrast, football imports average a younger age of 24.2 years. Sparta football distinctly positions itself as a development club, focusing on nurturing young talent to build competitive teams. In contrast, Sparta hockey pursues success at both domestic and European levels by leveraging older, more experienced players. This strategy aligns with their goal of achieving immediate competitiveness and securing titles in the highly competitive ice hockey leagues. AC Sparta football serves as a feeder to Western European teams, and HC Sparta both sends and receives with the elite NHL and KHL, yet neither team views exporting players as a talent drain (Agergaard, 2017; Marques, 2024). These migration exchanges further reinforce the respective Europeanization and Americanization of the two sports. The differing approaches underscore each club’s strategic priorities within their respective sports, with football prioritizing youth development and hockey emphasizing experience and immediate performance impact.

In the early days of their existence, both the football club AC Sparta Prague and the ice hockey club HC Sparta Prague featured foreign players on their rosters. However, the Second World War and the subsequent communist era significantly altered this practice. Since the fall of communism in 1989, the number of foreign players in both teams has steadily increased. Both clubs have also gained experience with foreign coaches, and there have been periods when foreign investors owned the clubs. Tolerance towards these foreign elements has grown over time, although the relationship with foreigners is still negatively influenced by the long history prior to 1989. As Dunning et al. (2004) noted, historical development and social processes within a society shape its resilience or acceptance of global influences. The lengthy history of communist rule and the subsequent transition to a market economy in Eastern Europe fostered a strong sense of national pride and cultural uniqueness, which in turn affects how sports are globalized.

The need to preserve cultural identity often leads to a selective incorporation of global elements, balancing modernization with the maintenance of traditional practices. While the communist era and the transition to a market economy have been associated with national pride and a frequent resistance to foreigners and internationalization, sports have played a significant role in changing these attitudes. In the realm of sports, people become more accustomed to foreigners and generally develop a positive attitude towards them. This is because foreign players and coaches bring high-quality performance, and foreign investors and fans bring financial resources to the clubs. Both AC Sparta Prague and HC Sparta Prague are the most popular clubs in the country, with large fan bases. As these clubs foster tolerance towards foreigners and internationalization, they positively influence the perception of globalization within the broader society. The clubs, especially AC Sparta Prague, actively engage with their fans in this regard and thus have a positive impact on how globalization and internationalization are accepted in Czech society.

One critical takeaway from these case studies is the need for sports managers to develop strategies that align with both global trends and local cultural identities. The success of Sparta’s football and ice hockey clubs demonstrates the importance of this balance. While embracing Americanization in hockey and Europeanization in football, the clubs have also maintained strong ties to their local fanbase, reinforcing the notion that cultural heritage can coexist with globalization. This highlights a key challenge in sports management: managing cultural diversity in a way that respects national identity while capitalizing on the benefits of internationalization.

In a broader context, the experiences of Sparta Prague underscore the relevance of managing cultural diversity as a competitive advantage in global sport. Successful internationalization strategies not only enhance on-field performance but also strengthen the financial sustainability of clubs through increased media attention, global fan engagement, and cross-border player movements. Furthermore, the selective incorporation of global elements, as seen in the strategies of both Sparta clubs, reflects the wider managerial imperative to adapt to cultural diversity while retaining unique organizational identities. The lessons from Sparta Prague illustrate the broader challenge in sports management of fostering inclusivity without diluting the essence of a club’s heritage (Richelieu and Webb, 2021). For sports managers worldwide, the ability to effectively manage this balance will be essential in building resilient, culturally rich, and globally competitive sports brands.

## Data Availability

The raw data supporting the conclusions of this article will be made available by the authors, without undue reservation.

## Footnotes

^1^England’s Premier League, Germany’s Bundesliga, Spain’s La Liga, Italy’s Serie A and France’s Ligue 1.
